# Dosimetric properties of equivalent‐quality flattening filter‐free (FFF) and flattened photon beams of Versa HD linear accelerator

**DOI:** 10.1120/jacmp.v17i3.6173

**Published:** 2016-05-08

**Authors:** Mukesh N. Meshram, Srimanta Pramanik, C.P. Ranjith, Saravana K. Gopal, Rishabh Dobhal

**Affiliations:** ^1^ CIMS Hospital Pvt. Ltd. Ahmedabad Gujarat India

**Keywords:** equivalent‐quality beam, flattening filter free, dosimetric properties, photon beam data, Versa HD linac

## Abstract

This study presents the basic dosimetric properties of photon beams of a Versa HD linear accelerator (linac), which is capable of delivering flattening filter‐free (FFF) beams with a beam quality equivalent to the corresponding flattened beams based on comprehensive beam data measurement. The analyzed data included the PDDs, profiles, penumbra, out‐of‐field doses, surface doses, output factors, head and phantom scatter factors, and MLC transmissions for both FFF and flattened beams of 6 MV and 10 MV energy from an Elekta Versa HD linac. The 6MVFFF and 10MVFFF beams had an equivalent mean energy to the flattened beams and showed less PDD variations with the field sizes. Compared with their corresponding flattened beams, $D_{\text{max}}$ was deeper for FFF beams for all field sizes; the ionization ratio variations with the field size were lower for FFF beams; the out‐of‐field doses were lower and the penumbras were sharper for the FFF beams; the off‐axis profile variations with the depths were lesser for the FFF beams. Further, the 6MVFFF and 10MVFFF beams had 35.7% and 40.9% less variations in output factor with the field size, respectively. The collimator exchange effect was reduced in the FFF mode. The head scatter factor showed 59.1% and 73.6% less variations, on average, for the 6MVFFF and 10MVFFF beams, respectively; the variations in the phantom scatter factor were also smaller. The surface doses for all beams increased linearly with the field size. The 6MVFFF and 10MVFFF beams had higher surface doses than the corresponding flattened beams for field sizes of up to $10\times 10{\text{cm}}^{2}$ but had lower surface doses for larger fields. Both FFF beams had lower average MLC transmissions than the flattened beams. The finding that the FFF beams were of equivalent quality to the corresponding flattened beams indicates a significant difference from the data on unmatched FFF beams.

PACS number(s): 87.56.bd, 87.55.Qr

## I. INTRODUCTION

The Versa HD linear accelerator (linac; Elekta AB, Stockholm, Sweden) is capable of delivering flattened and flattening filter‐free (FFF) beams. The device is configured with the Agility multileaf collimator assembly (Elekta AB) with a dedicated linac control system. The enhanced leaf and diaphragm speed helps to improve the treatment plan modulation with a high dose rate feature.

Modalities such as the IMRT and VMAT use varying fluence patterns and do not require flat homogeneous beams for plan optimization. Recently, many studies have reported the advantages of FFF beams over conventional flattened beams, such as a higher dose rate, reduced head scatter, smaller leakage, and lower out‐of‐field dose. The high dose rate is especially beneficial in SRS and SBRT, as well as in treatments that require organ motion management.

Various studies have reported the characteristics of FFF beams based on measurements^1^, ^2^, ^3^ and Monte Carlo simulation^4^, ^5^ from Varian linacs. The properties of FFF beams have also been summarized for Elekta^6^, ^7^, ^8^, ^9^ and Siemens^(10)^ linacs. However, most of the investigations were from linacs, which work as prototype for the quality‐matched FFF beam.

The removal of the flattening filter alone, without any further modifications in the beam control parameters, results in a higher dose rate and reduced beam quality.^1^, ^2^, ^3^, ^6^, ^7^, ^11^, ^12^ The beam quality can be increased to that of the corresponding flattened beam by matching the percentage depth dose (PDD) at 10 cm depth. Kragl et al.^(6)^ studied both 6 MV and 10 MV unmatched FFF beams and also investigated only the 6 MV matched FFF beam from an Elekta Precise linac. In this paper, we will follow the designs of the previous studies,^1^, ^2^, ^3^, ^6^, ^7^, ^8^, ^9^, ^10^, ^11^, ^13^, ^14^ particularly the Huang et al.^(10)^ study on the quality matched 6MVFFF beam provided by a Siemens linac, to provide medical physics community with our evaluation on the dosimetric differences between conventional flattened and equivalent‐quality FFF beams of lower and higher energies from Elekta Versa HD linac.

## II. MATERIALS AND METHODS

### A. Linear accelerator

The measurements of photon beam data were done on a Versa HD linac. We refer to the four photon beams as 6 MV, 6MVFFF, 10 MV, and 10MVFFF, with respective dose rates of 600 MU/min, 2000 MU/min, 500 MU/min, and 2400 MU/min.

The beam data were confirmed to be within the manufacturer's specifications and also followed the National Task Group recommendations^(15)^ for clinical operations. In the FFF mode, the quality‐matching process was done to achieve %dd(10) equivalent to those of the corresponding flattened beams at 10 cm depth for the 6MVFFF as well as 10MVFFF beam. Absolute calibration was done for the flattened as well as FFF beams. The beams were calibrated according to the TRS‐398 protocol;^(16)^ 1 MU corresponded to 1 cGy at a $D_{\text{max}}$ depth of 100 cm SSD and a field size of $10\times 10\;{\text{cm}}^{2}$.

### B. PDDs and profiles

The PDDs and profiles were acquired with a PTW MP3 water‐tank (PTW Freiburg, Germany) at 90 cm SSD for the following field sizes: $1\times 1{\text{cm}}^{2}$, $2\times 2\;{\text{cm}}^{2}$, $3\times 3\;{\text{cm}}^{2}$, $4\times 4\;{\text{cm}}^{2}$, $5\times 5\;{\text{cm}}^{2}$, $7\times 7\;{\text{cm}}^{2}$, $10\times 10\;{\text{cm}}^{2}$, $15\times 15\;{\text{cm}}^{2}$, $20\times 20\;{\text{cm}}^{2}$, $25\times 25\;{\text{cm}}^{2}$, $30\times 30\;{\text{cm}}^{2}$, $35\times 35\;{\text{cm}}^{2}$, and $40\times 40\;{\text{cm}}^{2}$.

The PDDs for field sizes ranging from $1\times 1\;{\text{cm}}^{2}$ to $4\times 4\;{\text{cm}}^{2}$ were acquired with a PinPoint chamber (product code 31014; PTW Freiburg); for field sizes greater than $4\times 4\;{\text{cm}}^{2}$, the PDDs were measured with a 0.125 cc ion chamber (product code 31010; PTW Freiburg). The PDD measurements were acquired at a step size of 0.1 cm, with the chamber position corrected to the effective point of measurement.^(16)^ The PDD variation at various depths, (0.5 mm, 100 mm, and 200 mm), and the $D_{\text{max}}$ value and beam quality associated with different energies were examined and compared between flattened and FFF beams. The dose falloff behavior of the depth‐dose curve can be evaluated by the ionization ratio of the doses at 20 cm and 10 cm depths (ionization ratio: $J=D_{20}/D_{10}$).

The profiles were measured at depth of $D_{\text{max}}$, 5 cm, 10 cm, and 20 cm. The profiles for field sizes ranging from $2\times 2{\text{cm}}^{2}$ to $5\times 5{\text{cm}}^{2}$ were acquired with a PinPoint chamber (product code 31014; PTW Freiburg); for field sizes greater than $5\times 5{\text{cm}}^{2}$, the measurements were done with a 0.125 cc ion chamber.

Pönisch et al.^(2)^ and Fogliata et al.^(13)^ proposed different methods to normalize the FFF profile by using the inflection point and shoulder point, respectively. We used the normalization method suggested by Fogliata and colleagues to compare the field widths, penumbras, and doses in the lateral regions of the profiles between flattened and FFF beams.

### C. Output factor and head scatter factor

The output factor ($S_{\text{cp}}$) is the ratio of the dose for a given field size to that of the reference field size of $10\times 10\;{\text{cm}}^{2}$. The $S_{\text{cp}}$ was measured in water phantom at 90 cm SSD and 10 cm depth for field sizes of $1\times 1\;{\text{cm}}^{2}$, $2\times 2\;{\text{cm}}^{2}$, $3\times 3\;{\text{cm}}^{2}$, $4\times 4\;{\text{cm}}^{2}$, $5\times 5\;{\text{cm}}^{2}$, $7\times 7\;{\text{cm}}^{2}$, $10\times 10\;{\text{cm}}^{2}$, $12\times 12\;{\text{cm}}^{2}$, $15\times 15\;{\text{cm}}^{2}$, $18\times 18\;{\text{cm}}^{2}$, $20\times 20\;{\text{cm}}^{2}$, $22\times 22\;{\text{cm}}^{2}$, $25\times 25\;{\text{cm}}^{2}$, $30\times 30\;{\text{cm}}^{2}$, $35\times 35\;{\text{cm}}^{2}$, $40\times 40\;{\text{cm}}^{2}$, $5\times 30\;{\text{cm}}^{2}$, and $30\times 5\;{\text{cm}}^{2}$. A PinPoint chamber was used for smaller field sizes of up to $5\times 5\;{\text{cm}}^{2}$; for field sizes of $7\times 7\;{\text{cm}}^{2}$ to $40\times 40\;{\text{cm}}^{2}$ and $5\times 30\;{\text{cm}}^{2}$ and $30\times 5\;{\text{cm}}^{2}$, a 0.125 cc ion‐chamber was used.

The head scatter factor ($S_{c}$) was measured in air isocentric conditions with a 0.125 cc ion chamber with buildup cap for square fields of $5\times 5{\text{cm}}^{2}$, $10\times 10{\text{cm}}^{2}$, $15\times 15{\text{cm}}^{2}$, $20\times 20{\text{cm}}^{2}$, $30\times 30{\text{cm}}^{2}$, and $40\times 40{\text{cm}}^{2}$, as well as for rectangular fields of $X\times 40\;{\text{cm}}^{2}$ and $40\times Y\;{\text{cm}}^{2}$, where X,Y=3,5,8,10,15,20, and 30 cm. The $S_{\text{cp}}$ and $S_{c}$ were normalized to the $10\times 10\;{\text{cm}}^{2}$ reference field. The phantom scatter factor ($S_{p}$) was then calculated by removing the head scatter from the total scatter.^(17)^

### D. Surface dose

The surface dose ($D_{s}$) was measured by using a Markus parallel plate chamber (product code 23343; PTW Freiburg) with a thin (0.025 mm thick) entrance window of water‐equivalent material.^14^, ^18^, ^19^, ^20^ Data were collected at 90 cm SSD for square fields ($F.S.=2\times 2\;{\text{cm}}^{2}$, $3\times 3\;{\text{cm}}^{2}$, $4\times 4\;{\text{cm}}^{2}$, $5\times 5\;{\text{cm}}^{2}$, $7\times 7\;{\text{cm}}^{2}$, $10\times 10\;{\text{cm}}^{2}$, $20\times 20\;{\text{cm}}^{2}$, and $30\times 30\;{\text{cm}}^{2}$). In this study, the surface dose is defined as the relative dose at a depth of 0.5 mm with respect to the dose at $D_{\text{max}}$. (20)

### E. Penumbra

The standard definition of penumbra for a conventional beam is not valid for an FFF beam. However, when the FFF beam is normalized to the same dose level as the flattened beam, as suggested by Fogliata et al.,^(13)^ it is possible to evaluate the penumbra as the difference in position between the 80% and 20% dose levels of the profile.

### F. Off‐axis ratio

The definition of flatness for evaluating the dose variation across the flattened region is not applicable to the FFF beam. However, when the FFF profile is renormalized, as previously described, a region within a certain percentage of the field can be used to define a parameter for evaluating the off‐axis profile variation for both beam modalities.^6^, ^10^, ^13^, ^15^ The off‐axis profile variations of both flattened and FFF beams for different field sizes at changing depths were quantified by using the off‐axis ratio (OAR). The OAR is defined as the ratio of the dose level at the edge of the field region to that at the beam central axis. We used the field region for field sizes $\geq 10\;{\text{cm}}^{2}$ as the area within 80% of the field and that for field sizes $<10\;{\text{cm}}^{2}$ as the area within 60% of the field.

### G. Out‐of‐field dose

The out‐of‐field doses for flattened and FFF beams were evaluated for field sizes of $5\times 5\;{\text{cm}}^{2}$ and $10\times 10\;{\text{cm}}^{2}$ at depths of 2 cm, 10 cm, and 20 cm. The half‐dose profiles up to 40 cm off the central axis were measured by placing the MP3 large‐water phantom asymmetrically. Measurements were done with the 0.125 cc ion chamber.

### H. Agility MLC transmission

The Agility MLC has 160 tungsten alloy leaves of 5 mm width resolution over the entire $40\times 40\;{\text{cm}}^{2}$ field at isocenter. The leaf has a nominal height of 90 mm and a speed of up to 3.5 cm/sec. The synchronization of the dynamic leaf guide movement with the individual leaf movement enhanced the leaf speed to up to 6.5 cm/sec. The leaves have flat edges with rounded ends and are tilted to reduce the overall transmission.^(21)^

The measurements were done by using a diode detector (product code 60016; PTW Freiburg) with the detector axis parallel to the central axis and an isocentric setup of SSD=95 cm and depth=5 cm. The MLC transmission was calculated as the average transmission reading to the isocentric reading of the $10\times 10\;{\text{cm}}^{2}$ reference field.^6^, ^7^, ^21^, ^22^

## III. RESULTS

### A. PDD

The PDD of FFF beams for the $10\times 10\;{\text{cm}}^{2}$ field size at 10 cm depth was matched to have within 0.5% difference from that of the corresponding flattened beams. The quality of FFF beams was matched to be equivalent to that of the corresponding flattened beams as specified in the Elekta customer acceptance test.^(23)^ The beam quality matching resulted in a shift of the $D_{\text{max}}$ for FFF beams to a deeper location compared with the corresponding flattened beams, as shown in Table 1.

The $D_{\text{max}}$ depths of FFF beams were deeper than those of the corresponding flattened beams for all the field sizes. For all beams, the $D_{\text{max}}$ shifted closer to the surface as the field size increased from $5\times 5\;{\text{cm}}^{2}$ to $40\times 40\;{\text{cm}}^{2}$. The shift was found to vary from 16 mm to 13 mm for the 6 MV beam and from 22 mm to 17 mm for the 10 MV beam; the corresponding values for the FFF beams varied from 18 mm to 16 mm and from 24 mm to 22 mm for the 6MVFFF and 10MVFFF beams, respectively.

**Figure 1:**
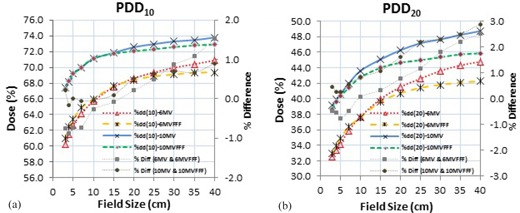
Comparison of PDDs at depth of (a) 10 cm and (b) 20 cm between flattened beams and FFF beams.

The variations in PDD associated with the increasing field sizes from $3\times 3\;{\text{cm}}^{2}$ to $40\times 40\;{\text{cm}}^{2}$ were observed to be 17.8%, 13.8%, 9.5%, and 8.5% for the 6 MV, 6MVFFF, 10 MV, and 10MVFFF beams, respectively, at a depth of 10 cm; the variations at a depth of 20 cm were 37.8%, 28.2%, 24.5%, and 18.3% for the 6 MV, 6MVFFF, 10 MV, and 10MVFFF beams, respectively. The 6MVFFF and 10MVFFF beams showed 22.5% and 10.5% less PDD variations with the field size at 10 cm depth compared with the corresponding flattened beams. Similarly, 25.4% and 25.3% less PDD variations at 20 cm depth were observed for the 6MVFFF and 10MVFFF beams, respectively, as shown in Figs. 1(a) and (b).

**Table 1:** Basic properties of depth‐dose curve.

*Energy*	$D_{max}$	*%dd (0.5 mm)*	*%dd(10)x*
6MVFFF	17 mm	27.5	68.43
10MVFFF	24 mm	23.9	73.24
6 MV	15 mm	27.2	67.92
10 MV	22 mm	22.0	72.95

$D_{\text{max}}=\text{depth of dose maximum}$; $\%\text{dd}({10)}_{X}=\text{percentage depth dose due to photon only at}\;10\;\text{cm depth}$.

The dose falloff behavior of FFF and flattened beams with changing field sizes can be evaluated by using the ionization ratio of the dose at 20 cm and 10 cm depths ($J=D_{20}/D_{10}$). Table 2 presents a summary of the ionization ratio $J=D_{20}/D_{10}$ for selected field sizes. The FFF beam depth‐dose curve shows a faster dose falloff compared with the conventional flattened beam. The variations in the ionization ratio with increasing field sizes from $3\times 3\;{\text{cm}}^{2}$ to $40\times 40\;{\text{cm}}^{2}$ were 17.0%, 11.9%, 13.7%, and 9.9% for the 6 MV, 6MVFFF, 10 MV, and 10MVFFF beams, respectively.

**Table 2:** Ionization ratio ($J=D_{20}/D_{10}$) for various field sizes.

*Field Size*	*6 MV*	*6MVFFF*	*% Diff*	*10 MV*	*10MVFFF*	*% Diff*
$3\times 3\;{\text{cm}}^{2}$	0.5397	0.5431	0.62	0.5805	0.5748	−0.98
$10\times 10\;{\text{cm}}^{2}$	0.5734	0.5699	−0.61	0.6131	0.6011	−1.95
$20\times 20\;{\text{cm}}^{2}$	0.6049	0.5953	−1.58	0.6373	0.6194	−2.81
$30\times 30\;{\text{cm}}^{2}$	0.623	0.6047	−2.93	0.6499	0.6246	−3.89
$40\times 40\;{\text{cm}}^{2}$	0.6316	0.6076	−3.80	0.6602	0.6315	−4.34

### B. Output factor and head scatter factor

**Figure 2:**
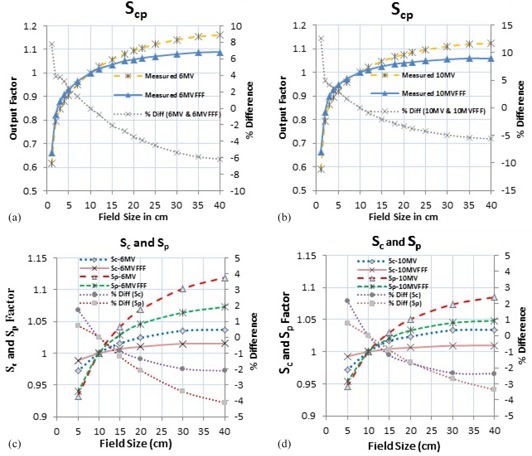
Total scatter factor are shown for 6 MV and 6MVFFF (a), 10 MV and 10MVFFF (b); head scatter factor and phantom scatter factor are shown for 6 MV and 6MVFFF (c) and 10 MV and 10MVFFF (d).

The variations in $S_{\text{cp}}$ for field sizes of $1\times 1\;{\text{cm}}^{2}$ to $40\times 40\;{\text{cm}}^{2}$ are plotted in Figs. 2(a) and (b). For field sizes of $3\times 3\;{\text{cm}}^{2}$ to $40\times 40\;{\text{cm}}^{2}$, the $S_{\text{cp}}$ varied from 0.847 to 1.160 and from 0.862 to 1.122 for the 6 MV and 10 MV flattened beams, respectively. The variations in the $S_{\text{cp}}$ associated with similar increase in the field sizes ranged from 0.879 to 1.088 and from 0.898 to 1.058 for the 6MVFFF and 10MVFFF beams, respectively, indicating that the 6MVFFF beam had 35.7% and the 10MVFFF beam had 40.9% less variations compared with the conventional flattened beams.

The changes in the $S_{\text{cp}}$ values because of the collimator exchange for rectangular fields was smaller for FFF beams. The $S_{\text{cp}}$ values changed by 0.91%, 0.46 %, 0.97%, and 0.46% for the 6 MV, 6MVFFF, 10 MV, and 10MVFFF beams, respectively, for $5\times 30\;{\text{cm}}^{2}$ and $30\times 5\;{\text{cm}}^{2}$ rectangular fields, the $S_{\text{cp}}$ values were found to be 49.4% and 52.5% less compared with 6 MV and 10 MV flattened beams, respectively.

For symmetrical fields of $5\times 5\;{\text{cm}}^{2}$ to $40\times 40\;{\text{cm}}^{2}$, the head scatter factor ($S_{c}$) ranged from 0.972 to 1.037 for the 6 MV and from 0.971 to 1.034 for the 10 MV flattened beam. The $S_{c}$ varied from 0.988 to 1.015 for the 6MVFFF and from 0.992 to 1.009 for the 10MVFFF beams, respectively. Also, the variations in the phantom scatter factor ($S_{p}$) were observed to be 20%,

14.7%, 14.2%, and 10% for the 6 MV, 10 MV, 6MVFFF, and 10MVFFF beams, respectively; these were 29.0% and 32.0% less than in the 6MVFFF and 10MVFFF beams respectively, with similar changes in the field sizes. Figs. 2(c) and (d) show the variations in $S_{c}$ and $S_{p}$ with the changing field sizes. For rectangular fields, the FFF beams had smaller $S_{c}$ variations compared with the corresponding flattened beams.

The 6MVFFF and 10MVFFF beams for both square and rectangular fields had, on average, 59.1% and 73.6% smaller $S_{c}$ variations, respectively, compared with the corresponding flattened beams.

### C. Surface dose

**Figure 3:**
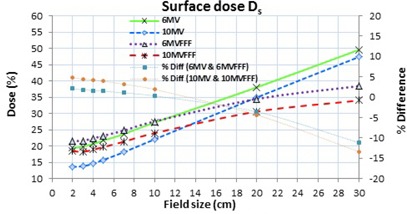
Relative surface doses ($D_{s}$) for flattened and FFF beams with changing field sizes.

The surface doses for both flattened and FFF beams were found to increase linearly with the field size, as shown in Fig. 3. The FFF beams had marginally higher surface doses compared with the corresponding flattened beams for field sizes ranging from $2\times 2\;{\text{cm}}^{2}$ to $10\times 10\;{\text{cm}}^{2}$, but had lower surface doses for larger field sizes. The relative surface dose with increasing field sizes from $2\times 2\;{\text{cm}}^{2}$ to $30\times 30\;{\text{cm}}^{2}$ was increased by 30.1%, 16.9%, 33.7%, and 15.6% for the 6 MV, 6MVFFF, 10 MV, and 10MVFFF beams, respectively, indicating that FFF beams had smaller surface dose variations with the field size compared with the corresponding flattened beams.

### D. Penumbra

**Figure 4:**
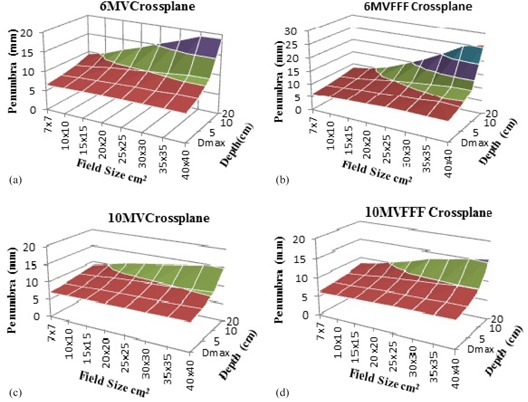
Transverse penumbral widths (mm) for 6 MV (a), 6MVFFF (b), 10 MV (c), and 10MVFFF (d) as function of field size and depth at 90 cm SSD.

The penumbras measured for different field sizes at different depths were evaluated after renormalization, as previously described; Table 3 shows the measurements obtained in the selected conditions. The penumbra of the 6MVFFF beams was observed to be sharper than that of the 6 MV flattened beam for field sizes ranging from $2\times 2\;{\text{cm}}^{2}$ to $10\times 10\;{\text{cm}}^{2}$ at all measured depths. Widening of penumbra with increasing field size was observed for all beams. However, this effect was less pronounced for the flattened beams; thus, the penumbral width difference between flattened and FFF beams gradually diminished and eventually resulted in a wider penumbra for the 6MVFFF beam compared with the 6 MV flattened beam for field sizes ranging from $15\times 15\;{\text{cm}}^{2}$ to $40\times 40\;{\text{cm}}^{2}$ at all measured depths. The same widening behavior of the penumbra was also observed for both the 10 MV flattened and the 10MVFFF beams, but the percentage variation of the penumbra with increasing field size was lower for the high‐energy than for the low‐energy beams under investigation at all measured depths, as shown in Fig. 4. This resulted in a sharper penumbra for the 10MVFFF beam compared with the 10 MV flattened beam in all measured conditions, except for field sizes ranging from $25\times 25\;{\text{cm}}^{2}$ to $40\times 40\;{\text{cm}}^{2}$ at a depth of 20 cm.

**Table 3:** Transverse penumbral width (mm) for selected field sizes at $D_{\text{max}}$ and 10 cm depth.

*Depth*	*F.S.*	$2\times 2\;cm^{2}$	$10\times 10\;cm^{2}$	$15\times 15\;cm^{2}$	$20\times 20\;cm^{2}$	$35\times 35\;cm^{2}$
$D_{\text{max}}$	6 MV	3.9	6.65	6.8	6.9	7.0
	6MVFFF	3.4	6.4	6.8	7.0	7.3
	10 MV	4.0	7.2	7.3	7.4	7.6
	10MVFFF	3.5	6.4	6.6	6.7	7.1
10 cm	6 MV	4.6	8.4	9.1	9.5	10.9
	6MVFFF	4.1	7.9	8.9	9.9	12.0
	10 MV	4.7	8.5	9.0	9.3	10.2
	10MVFFF	4.1	7.7	8.3	8.7	9.9

### E. Off‐axis ratio

Table 4 presents the off‐axis ratios (OAR) for different field sizes at changing depths of 90 cm SSD for the 10 MV flattened and 10MVFFF beams. The FFF beams showed smaller variations in beam quality along the off‐axis with increasing depth compared with the corresponding flattened beams. The OAR variations in the $20\times 20\;{\text{cm}}^{2}$ cross‐plane profiles with increasing depth were observed to be 8.4% and 6.4% for the 6 MV and 10 MV beams, and 2.8% and 3.3% for the 6MVFFF and 10MVFFF beams, respectively.

**Table 4:** Off‐axis‐ratio for selected field sizes measured at different depths (D) and 90 cm SSD.

*Field Size (cm^2^)*	*Energy*	$D=D_{\text{max}}$	D=5 cm	D=10 cm	D=20 cm
2×2	10 MV	0.858	0.861	0.872	0.879
	10MVFFF	0.915	0.913	0.912	0.912
4×4	10 MV	0.970	0.966	0.963	0.964
	10MVFFF	0.947	0.943	0.941	0.943
10×10	10 MV	0.986	0.973	0.958	0.942
	10MVFFF	0.827	0.818	0.808	0.803
15×15	10 MV	1.007	0.990	0.973	0.944
	10MVFFF	0.748	0.741	0.730	0.721
20×20	10 MV	1.011	1.004	0.981	0.946
	10MVFFF	0.669	0.664	0.656	0.647
25×25	10 MV	1.020	1.014	0.992	0.947
	10MVFFF	0.607	0.602	0.595	0.582
30×30	10 MV	1.042	1.029	1.006	0.951
	10MVFFF	0.550	0.545	0.542	0.533
35×35	10 MV	1.047	1.034	1.007	0.951
	10MVFFF	0.502	0.497	0.489	0.486
40×40	10 MV	1.049	1.034	1.007	0.952
	10MVFFF	0.459	0.456	0.452	0.443

### F. Out‐of‐field dose

**Figure 5:**
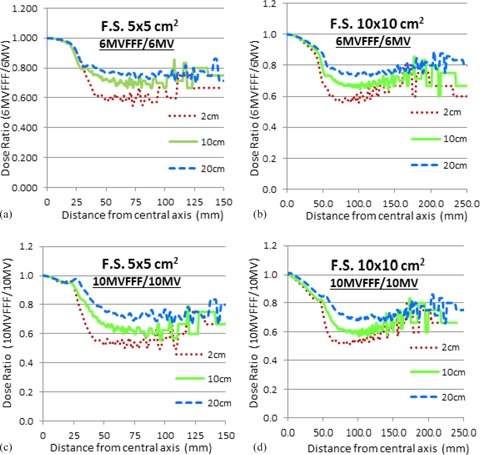
Out‐of‐field dose ratios are shown for field sizes $5\times 5\;{\text{cm}}^{2}$ and $10\times 10\;{\text{cm}}^{2}$ for 6MVFFF/6 MV (a), (b) and 10MVFFF/10 MV (c), (d) measured at 2 cm, 10 cm, and 20 cm depth and 90 cm SSD.

The out‐of‐field doses of flattened and FFF beams for $5\times 5\;{\text{cm}}^{2}$ and $10\times 10\;{\text{cm}}^{2}$ field sizes at three different depths are plotted in Fig. 5. In all measurement conditions, the FFF beams were observed to have lower out‐of‐field doses compared with the corresponding flattened beams. Also, with increasing depth, a smaller difference between the dose deposition of the FFF and flattened beams was observed.

### G. Agility MLC transmission

The mean interleaf leakages were found to be 0.5% for both the 6 MV and 10 MV flattened beams; for the 6MVFFF and 10MVFFF beams, the mean interleaf leakages were 0.3% and 0.2%, respectively. The mean intraleaf leakages were 0.4% and 0.5% for the 6 MV and 10 MV flattened beams, respectively; for both the 6MVFFF and 10MVFFF beams, the mean intraleaf leakage was 0.2%. In all measurement positions, the average MLC transmission for FFF beams was found to be lower than that of the corresponding flattened beams.

## IV. DISCUSSION

To our knowledge, this is the first study to report on the dosimetric properties of flattened and FFF beams with equivalent‐beam qualities from a Versa HD linac that is commercially available for clinical use. The comparative dosimetric behaviors of flattened and FFF beams observed in this work are consistent with the data reported by previous studies. However, the equivalent‐quality FFF beams showed some unique characteristics in relation to the energy‐unmatched FFF beams.

As described in previous studies, a twofold increased dose rate was observed after the removal of the flattening filter without any changes in the beam control parameters. However, in the present work, as the FFF beam quality was matched to be equivalent to the corresponding flattened beam, the dose rate of the 6MVFFF beam was found to increase 3.3 times, higher than the dose rate without beam tuning. Similarly, the dose rate of the 10MVFFF beam was increased 4.8 times after beam quality matching. Huang et al.^(10)^ reported that the dose rate of the eqUF 6 MV unflat beam was five times higher than that of the 6 MV flattened beam. In contrast with previously reported data regarding the shift of $D_{\text{max}}$ toward the surface for energy‐unmatched FFF beams, the $D_{\text{max}}$ of equivalent‐quality FFF beams was observed to be deeper compared with the corresponding flattened beams. This effect was explained by Huang et al.,^(10)^ who reported that the $D_{\text{max}}$ shift was influenced by two competitive processes: the increased contribution of low‐energy photons due to the removal of the flattening filter (upstream shift of $D_{\text{max}}$), and the increased number of penetrating photons because of the increased beam quality (downstream shift of $D_{\text{max}}$). The combined effect of these two competitive processes results in a deeper $D_{\text{max}}$ for equivalent‐quality FFF beams. The decrease in $D_{\text{max}}$ with increasing field size is expected due to the increased head scatter with the field size and is consistent with previously reported data supporting the explanation of Vassiliev et al.^(3)^ and Kragl et al.^(6)^ for all beams.

The dosimetric behaviors of the depth‐dose curves, scatter factors, and surface doses for the field sizes observed in the present study are consistent with previously reported data. However, the observed decrease in the $S_{c}$ variation with the field size is higher than that reported by Kragl et al.^(6)^ and Cashmore^(7)^ for the Elekta Precise linac and by Huang et al.^(10)^ for the Siemens Oncor linac. The reduced collimator exchange effects because of the removal of the flattening filter are comparable with the previously published data by Cashmore^(7)^ and Kragl et al.^(6)^

The linear variation in surface dose with the field size is consistent with the findings reported by Wang et al.^(14)^ for the Varian linac, in which FFF beams were not matched with flattened beams. In the present study, because the FFF beams had an equivalent mean energy with an increased number of penetrating photons compared with unmatched FFF beams, the observed difference between flattened and FFF beams was marginal. The increased scatter contribution of flattened beams with the field size resulted in a higher surface dose for the flattened beams compared with the FFF beams for larger field sizes.

The penumbras of FFF beams for smaller field sizes were found to be sharper than those of flattened beams due to the reduced head scatter; this is consistent with the previously reported data by Hrbacek et al.^(1)^ A wider penumbra was observed for larger field sizes, which could be explained by the higher rate of penumbra widening with increasing field sizes for FFF beam.

This may be due to the requirement of a higher normalization factor for a more pronounced peak of FFF profiles and results in a larger penumbra for large field sizes of FFF beams. The lower OAR variations and out‐of‐field doses for FFF beams are related to the reduced head scatter and comparable with other published data.^(24)^

## V. CONCLUSIONS

The basic dosimetric properties of photon beams of the Versa HD linear accelerator were obtained. The concept of beam quality matching enhanced the efficiency of FFF beams in clinical implementation. The dosimetric study results indicate a significant difference from the data on unmatched FFF beam. In particular, the reduced surface dose with a deeper $D_{\text{max}}$ and the significant reduction in the variation of the head scatter factor with the field sizes results in a more beneficial effect on skin sparing.

## ACKNOWLEDGMENTS

The authors wish to thank S. Bargavan, Physicist (Elekta Medical System India Pvt. Ltd.) for his suggestions related to this study.

## COPYRIGHT

This work is licensed under a Creative Commons Attribution 4.0 International License.

## Supporting information

Supplementary Material

Supplementary Material

Supplementary Material
