# Supplementary figures and images for: Dosimetric properties of equivalent‐quality flattening filter‐free (FFF) and flattened photon beams of Versa HD linear accelerator
Source: J Appl Clin Med Phys. 2016 May 8;17(3):358–70. doi: 10.1120/jacmp.v17i3.6173 (PMC5690903; doi:10.1120/jacmp.v17i3.6173)

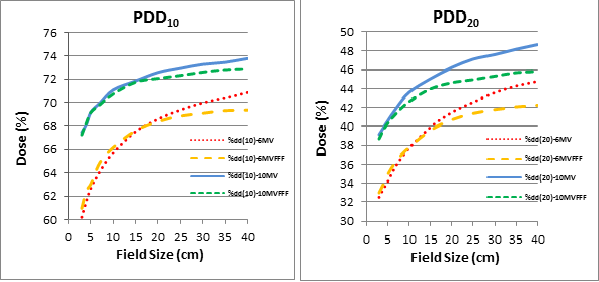

Supplement: Supplementary file 1 — Supplementary Material [file ACM2-17-358-s001.png]

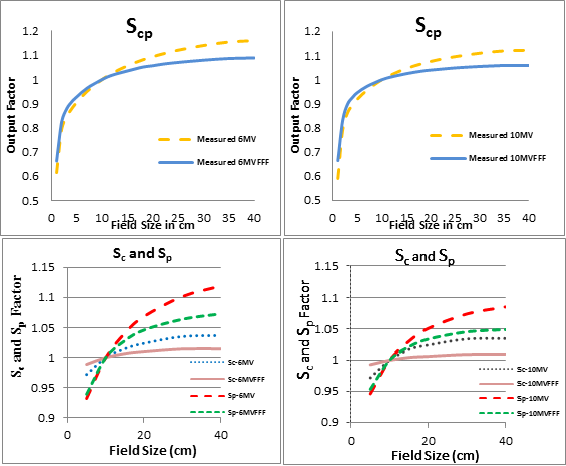

Supplement: Supplementary file 2 — Supplementary Material [file ACM2-17-358-s002.png]

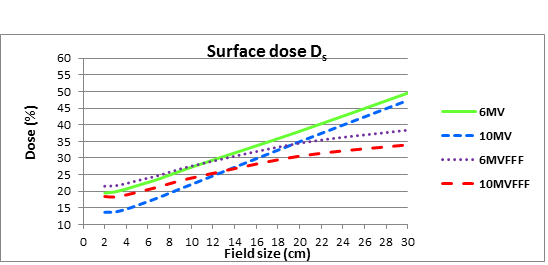

Supplement: Supplementary file 3 — Supplementary Material [file ACM2-17-358-s003.png]
